# Supplementary material for: Common dietary emulsifiers promote metabolic disorders and intestinal microbiota dysbiosis in mice
Source: Commun Biol. 2024 Jun 20;7:749. doi: 10.1038/s42003-024-06224-3 (PMC11190199; doi:10.1038/s42003-024-06224-3)
Supplement: Supplementary file 2 — Supplementary Information [file 42003_2024_6224_MOESM2_ESM.pdf]

Supplementary Information

Common dietary emulsifiers promote metabolic disorders and intestinal microbiota dysbiosis in mice

Supplementary Figures

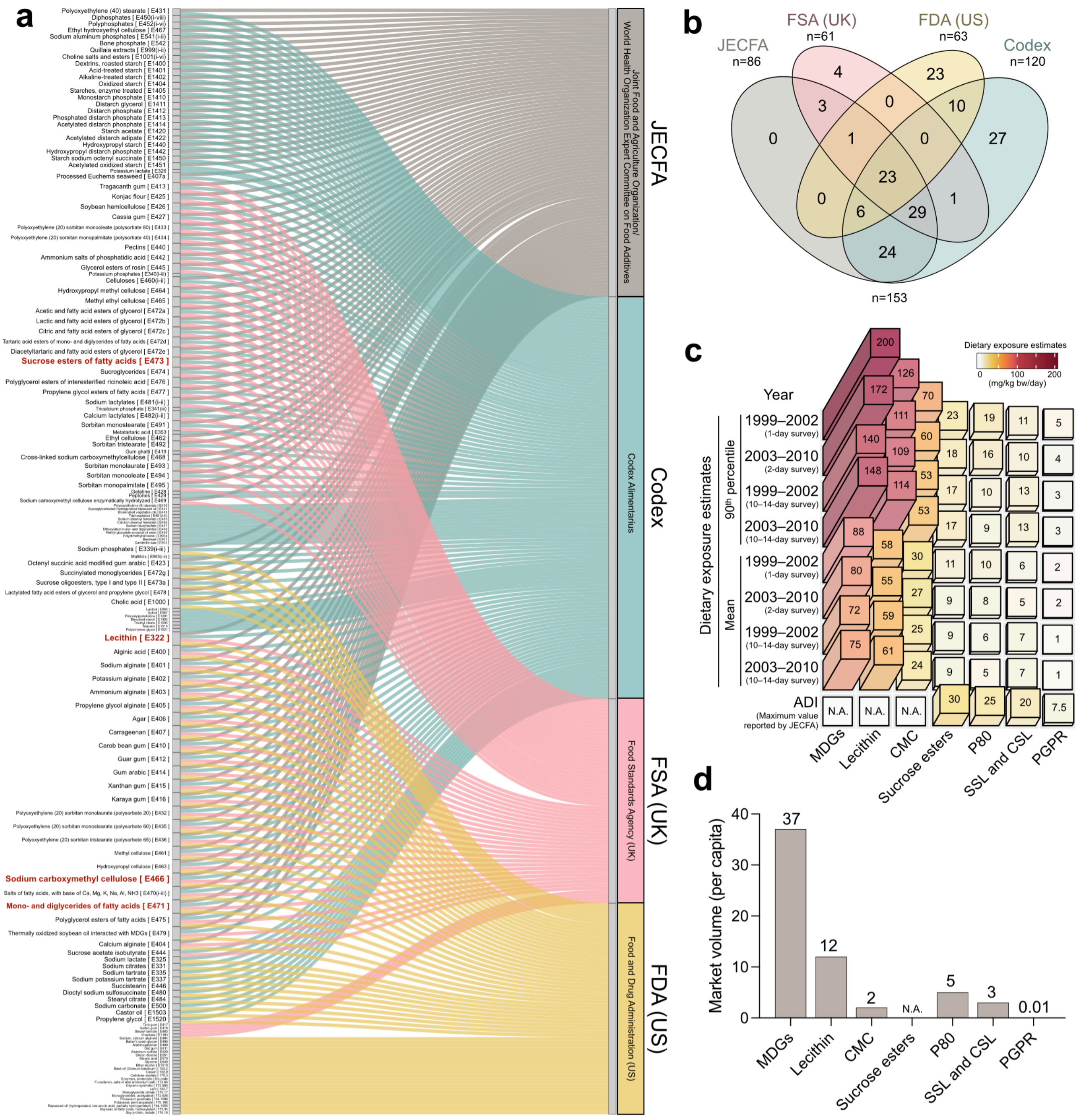

Supplementary Figure 1. Dietary exposure to common food emulsifiers and market volume of additives classified as food emulsifiers according to regulatory bodies involved in food additive legislation. a) Food additives are categorized as emulsifiers according to regulatory bodies that govern food additive usage, such as JECFA, Codex, FSA (UK), and FDA (US)[1]. **b)** Venn diagram showing the overlap between food emulsifier classifications by different organizations[1]. **c)** Dietary exposure to seven common emulsifiers compared with ADI[2] and **d)** market volume of these emulsifiers[2]. JECFA: Joint Food and Agriculture Organization/World

Health Organization Expert Committee on Food Additives; Codex: Codex Alimentarius; FSA: Food Standards Agency; UK: United Kingdom; FDA: Food and Drug Administration; US: United States; MDG: mono- and diglyceride; CMC: carboxymethylcellulose; P80: polysorbate 80; SSL: sodium stearoyl lactylate; CSL: calcium stearoyl lactylate; PGPR: polyglycerol polyricinoleate.

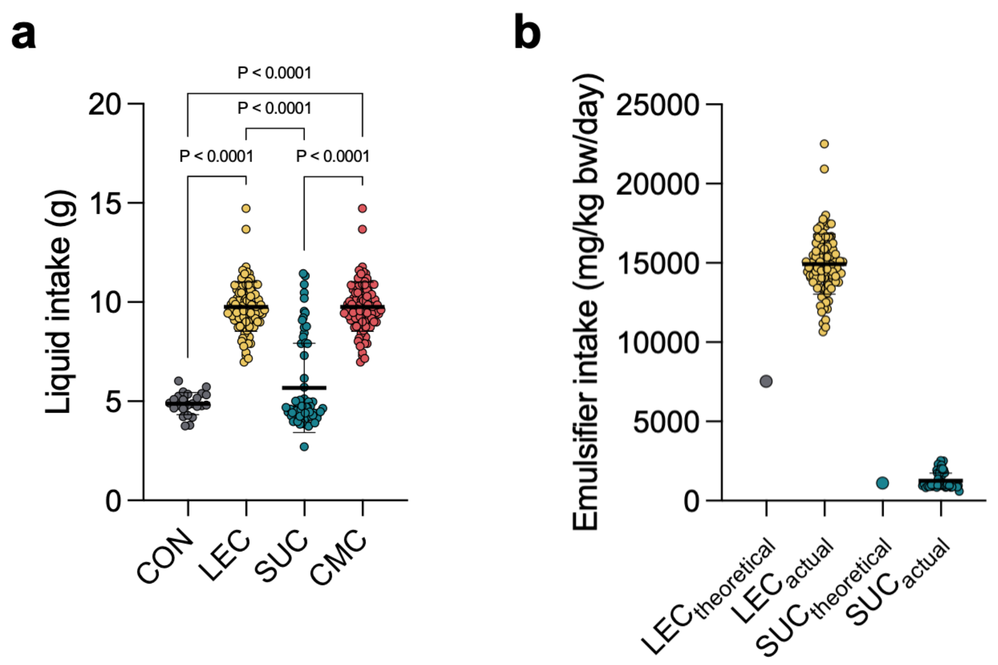

Supplementary Figure 2. Intake of dietary emulsifiers lecithin, sucrose fatty acid esters, and carboxymethylcellulose in mice. a) Liquid intake. **b)** Comparison of theoretical and actual emulsifier intake. Mice were supplemented with or without different emulsifiers in drinking water for 17 weeks. Bars express the mean ± s.d. Statistical analyses were performed using one-way ANOVA with the Tukey's range test for comparisons shown as exact P-values. CON: control; LEC: lecithin; SUC: sucrose fatty acid esters; CMC: carboxymethylcellulose.

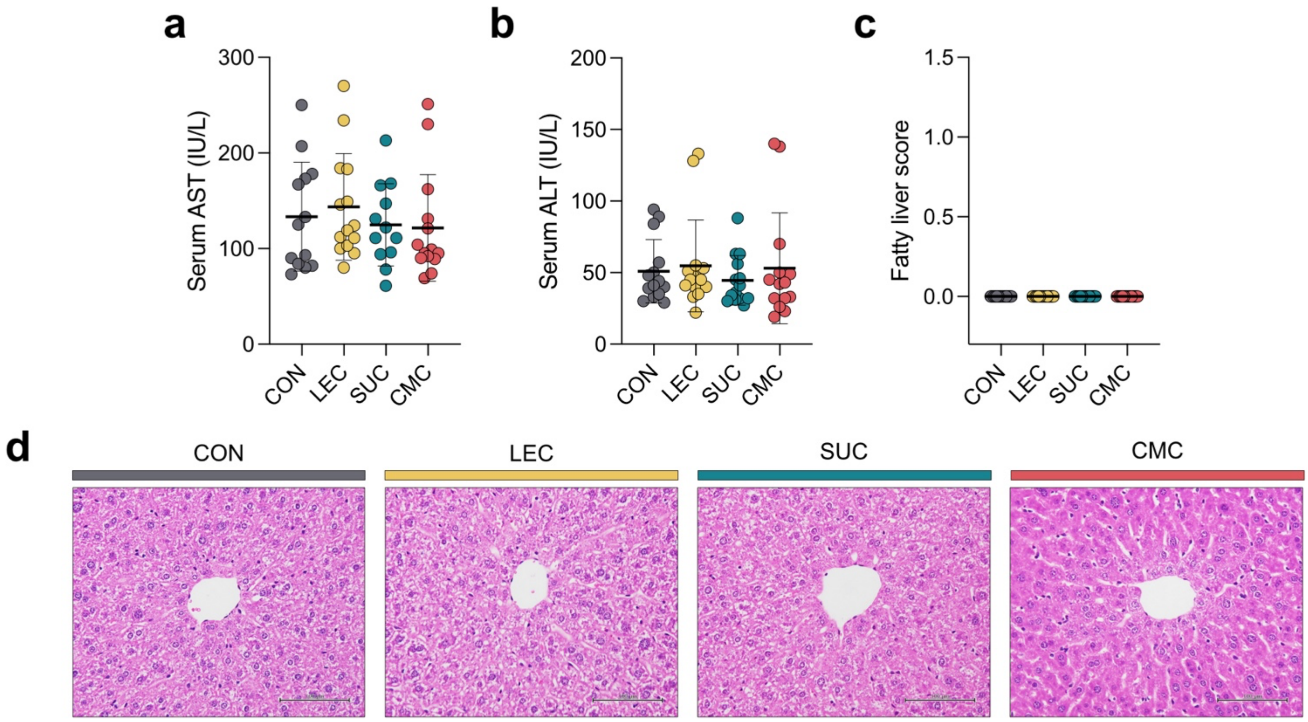

Supplementary Figure 3. Effect of dietary emulsifiers lecithin, sucrose fatty acid esters, and carboxymethylcellulose on liver-related biomarkers. a) Serum aspartate aminotransferase (AST), **b)** alanine aminotransferase (ALT) levels, **c)** fatty liver scores, and **d)** representative histological features of H&E-stained liver sections of each group (scale bar is 100 μm). Mice were supplemented with or without different emulsifiers in drinking water for 17 weeks. Dot plots are expressed as the mean ± s.d. (n = 14–15 per group). Statistical analyses were performed using one-way ANOVA with Tukey's range test for comparisons and shown as exact P-values. CON: control group; LEC: lecithin group; SUC: sucrose fatty acid esters group; CMC: carboxymethylcellulose group.

Emulsifier	The dose of emulsifiers administered to mice mimics human exposure levels (10X)		Emulsifier doses for 3T3-L1 cell (Translation from mouse dose)	
	mg/kg bw/day	mg [for 28 g mouse/day]	1:100 mg/mL	1:1000 mg/mL
LEC 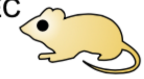 Lecithin	7523	210.644	2.1064	0.2106
SUC 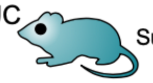 Sucrose ester	1110	31.08	0.3108	0.0311
CMC 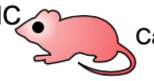 Carboxymethylcellulose	3486 (Calculated based on actual intake)	97.6	0.9760	0.0976

Supplementary Figure 4. The conversion of emulsifier doses administered to mice into doses suitable for the insulin resistance model in 3T3-L1 adipocytes. Illustrations in this figure were created with Keynote.

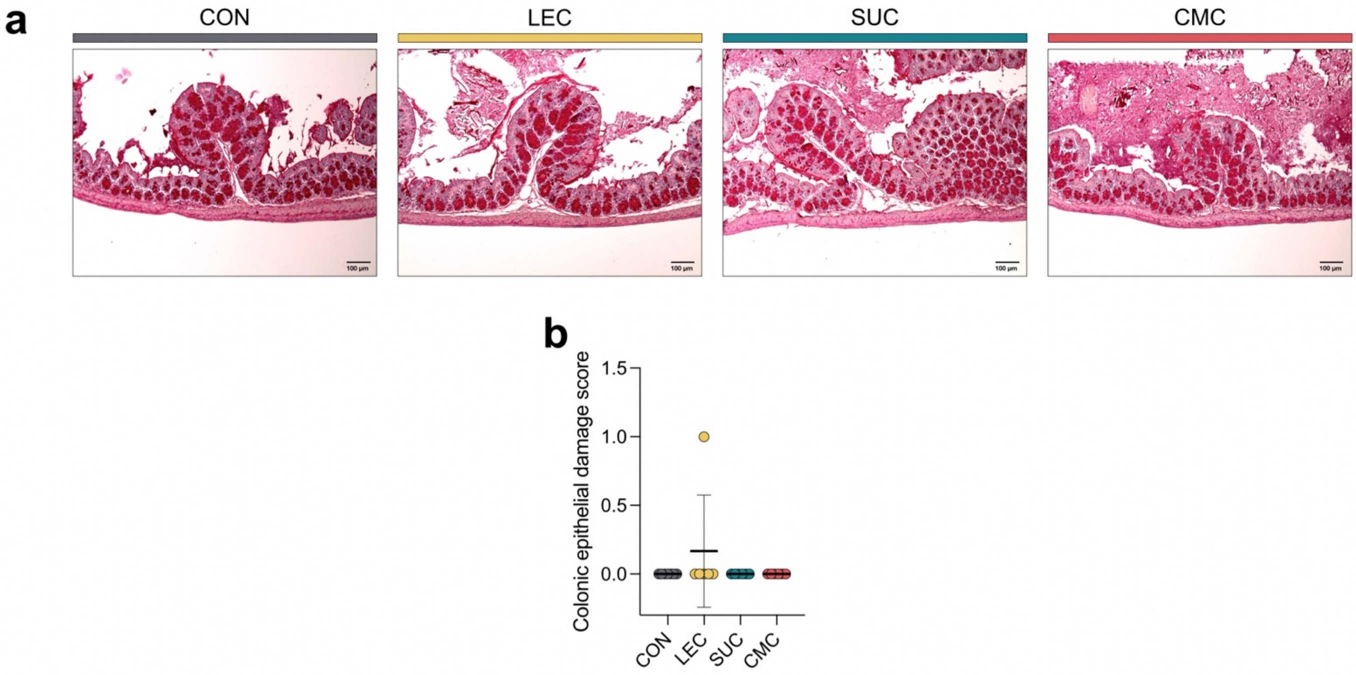

Supplementary Figure 5. Effect of dietary emulsifiers lecithin, sucrose fatty acid esters, and carboxymethylcellulose on colon histology. a) Representative histological features of Periodic acid-Schiff's (PAS)-stained colon sections (images are 100× magnification) and **b)** colonic epithelial damage histological scores of each group. Mice were supplemented with or without different emulsifiers in drinking water for 17 weeks. Dot plots are expressed as the mean ± s.d. Statistical analyses were performed using one-way ANOVA with Tukey's range test for comparisons. CON: control group; LEC: lecithin group; SUC: sucrose fatty acid esters group; CMC: carboxymethylcellulose group.

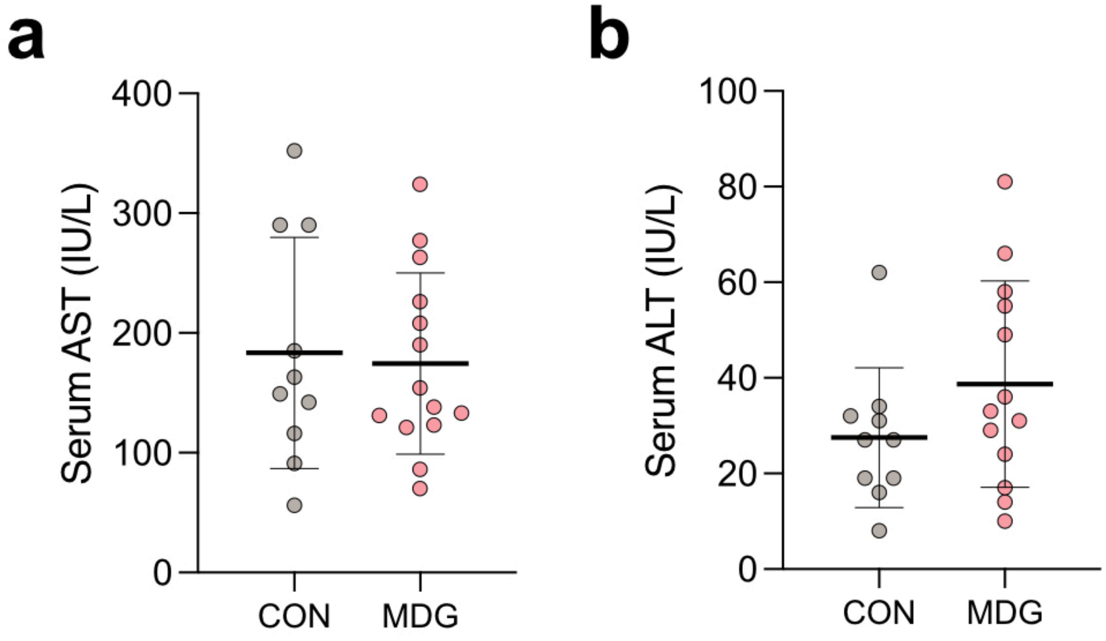

Supplementary Figure 6. Effect of monoglycerides and diglycerides (MDG) on liver-related biomarkers. a) Serum aspartate aminotransferase (AST) and **b)** alanine aminotransferase (ALT) levels. Mice were fed control or MDG diet for 14 weeks. Dot plots are expressed as the mean ± s.d. Statistical analyses were performed using an unpaired two-tailed Student's t-test for comparisons (CON vs. MDG). CON: control group; MDG: monoglycerides and diglycerides group.

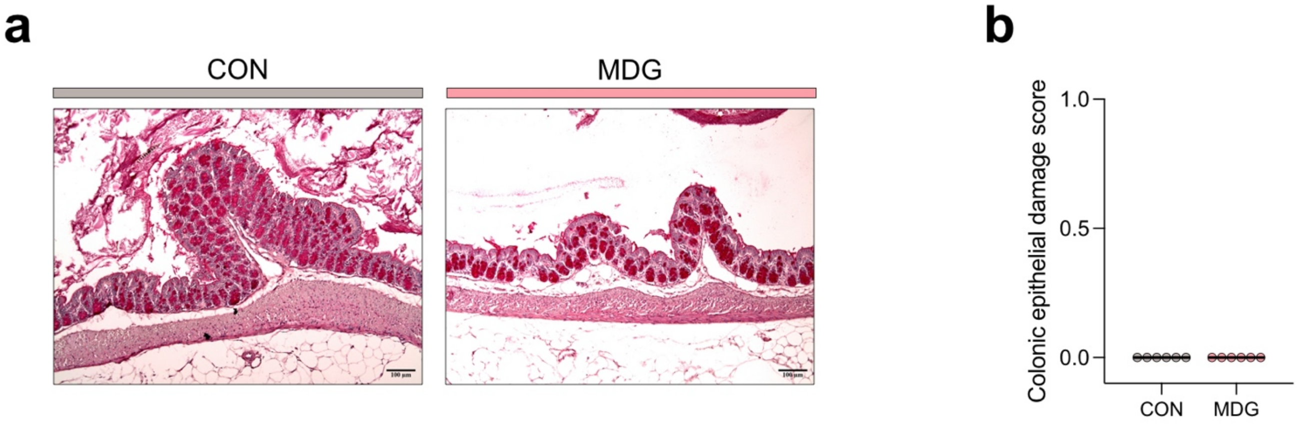

Supplementary Figure 7. Effect of monoglycerides and diglycerides (MDG) on colon histology. a) Representative histological features of Periodic acid-Schiff's (PAS)-stained colon sections (100× magnification), and **b)** colonic epithelial damage histological scores in each group. Dot plots are expressed as the mean ± s.d. Statistical analyses were performed using an unpaired two-tailed Student's t-test for comparisons (CON vs. MDG) and shown as exact P-value. CON: control group; MDG: monoglycerides and diglycerides group.

Supplementary Table

Supplementary Table 1. Comprehensive information on the association of gut microbiota taxa whose abundance changed in groups treated with hydrophilic/lipophilic emulsifiers with health- and disease-related factors.

Taxon	Health/disease-related factors	References
Akkermansia muciniphila	Pasteurized *A. muciniphila* supplementation enhances insulin sensitivity and decreases insulinemia, plasma total cholesterol levels, and obesity in overweight/obese insulin-resistant volunteers.	3
Atopostipes	*Atopostipes* abundance is enriched in mice with carbon tetrachloride-induced hepatic injury.	4
Blautia	*Blautia* abundance is enriched in patients with NASH, and is associated with increased lipopolysaccharide levels, visceral fat accumulation and obesity in adults, and increased blood insulin levels in children.	5-7
Collinsella	Low dietary fiber consumption increases *Collinsella* abundance, and *Collinsella* abundance correlates with circulating insulin levels in overweight/obese pregnant women.	8
Coriobacteriaceae UCG–002	*Coriobacteriaceae* UCG–002 exhibits anti-inflammatory function, and augmented *Coriobacteriaceae* UCG–002 abundance increases the beneficial bacterial metabolite short-chain fatty acid levels.	9
Clostridium sensu stricto 1	*Clostridium sensu stricto 1* abundance is increased in patients with duodenal strictures.	10
Desulfovibrio	*Desulfovibrio* plays a vital role in NAFLD pathogenesis by increasing intestinal permeability and hepatic CD36 expression.	11
Dubosiella	*Dubosiella* relative abundance is decreased in mice with DSS-induced colitis, having potential as ulcerative colitis amelioration bacteria.	12
Faecalibaculum rodentium	*F. rodentium* stimulates epithelial proliferation and turnover by dampening retinoic acid generation that helps intestinal eosinophil survival.	13
Enterobacter aerogenes and *Enterobacter cloacae*	Enterobacteriaceae is associated with inflammatory bowel disease pathogenesis and progression. *E. aerogenes* and *E. cloacae* are found in several outbreaks of hospital-acquired infections.	14,15
Enterorhabdus	*Enterorhabdus* relative abundance is increased in patients with prediabetes.	16

Eubacterium coprostanoligenes	*Eubacterium coprostanoligenes* group is largely found in patients with homocystinuria.	17
[Eubacterium] xylanophilum group	*[Eubacterium] xylanophilum* group abundance is enriched in mice with high salt-induced hypertension.	18
Jeotgalicoccus	*Jeotgalicoccus* is positively correlated with insulin concentration in diabetic rats.	19
Lachnoclostridium	*Lachnoclostridium* is associated with obesity, and highly abundant *Lachnoclostridium* is linked to decreased circulating acetate levels, which are associated with increased visceral fat in a large population-based cohort.	20,21
Lachnospiraceae NK4A136 group	*Lachnospiraceae* NK4A136 group is a potential probiotic whose abundance is reduced in high-fat diet-fed mice.	22
Lachnospiraceae UCG-006	*Lachnospiraceae* UCG-006 abundance is increased in high-fat diet-fed mice.	23
Muribaculaceae	*Muribaculaceae* abundance is enriched in lean mice and potentially involved in complex carbohydrate degradation.	24,25
Muribaculum	*Muribaculum* potentially maintains mouse gut homeostasis.	26
Olsenella	*Olsenella* abundance is enriched in lean people.	27
Oscillibacter	*Oscillibacter* abundance is enriched in diabetic mice fed a high-fat carbohydrate-free diet.	28
Parabacteroides distasonis	*P. distasonis* alleviates obesity and metabolic dysfunction by producing succinate and secondary bile acid.	29
Parasutterella	*Parasutterella* potentially plays a role in bile acid maintenance and cholesterol metabolism, and is associated with improved low-density lipoprotein levels in healthy individuals.	30,31
Staphylococcus aureus	*S. aureus* easily colonizes the infant's intestine due to a poorly competitive gut microbiota community.	32
*Streptococcus pyogenes*is	*S. pyogenes*is causes both non-invasive and invasive illnesses, including nonsuppurative sequelae.	33
Streptococcus gallolyticus	*S. gallolyticus* colonization is associated with colorectal cancer occurrence.	34
Turicibacter	*Turicibacter* is more abundant in lean than in obese rodents, and is a potential anti-inflammatory taxon.	35

Supplementary Table 2. Nutrition facts of the AIN-93M and new (MDG) diets (Research Diets, Inc.).

D10012M and New Diet

AIN-93M Mature Rodent Diet and Same with Soybean Oil Replaced by MDG/kg

Product #	D10012M		New Diet	
	gm%	*kcal%*	gm%	*kcal%*
Protein	14	*15*	14	*15*
Carbohydrate	73	*76*	73	*76*
Fat	4	9	4	9
Total		*100*		*100*
kcal/gm	3.8		3.8	
**Ingredient**	**gm**	***kcal***	**gm**	***kcal***
Casein	140	*560*	140	*560*
L-Cystine	1.8	*7.2*	1.8	*7.2*
Corn Starch	495.692	*1982.768*	495.692	*1982.768*
Maltodextrin 10	125	*500*	125	*500*
Sucrose	100	*400*	100	*400*
Cellulose, BW200	50	*0*	50	*0*
Soybean Oil	40	*360*	0	*0*
t-Butylhydroquinone	0.008	*0*	0.008	*0*
Mineral Mix S10022M	35	*0*	35	*0*
Vitamin Mix V10037	10	*40*	10	*40*
Choline Bitartrate	2.5	*0*	2.5	*0*
**Mono and Diglycerides (MDG)**	0	*0*	**40**	*360*
**Total**	***1000***	***3850***	***1000.00***	***3850***

Supplementary References

1 Cox, S., Sandall, A., Smith, L., Rossi, M. & Whelan, K. Food additive emulsifiers: a review of their role in foods, legislation and classifications, presence in food supply, dietary exposure, and safety assessment. *Nutr Rev* **79**, 726-741, doi:10.1093/nutrit/nuaa038 (2021).

2 Shah, R., Kolanos, R., DiNovi, M. J., Mattia, A. & Kaneko, K. J. Dietary exposures for the safety assessment of seven emulsifiers commonly added to foods in the United States and implications for safety. *Food Addit Contam Part A Chem Anal Control Expo Risk Assess* **34**, 905-917, doi:10.1080/19440049.2017.1311420 (2017).

3 Depommier, C. *et al.* Supplementation with *Akkermansia muciniphila* in overweight and obese human volunteers: a proof-of-concept exploratory study. *Nat Med* **25**, 1096-1103, doi:10.1038/s41591-019-0495-2 (2019).

4 Xu, Y. *et al. Flammulina velutipes* polysaccharides modulate gut microbiota and alleviate carbon tetrachloride-induced hepatic oxidative injury in mice. *Front Microbiol* **13**, 847653, doi:10.3389/fmicb.2022.847653 (2022).

5 Del Chierico, F. *et al.* Gut microbiota profiling of pediatric nonalcoholic fatty liver disease and obese patients unveiled by an integrated meta-omics-based approach. *Hepatology* **65**, 451-464, doi:10.1002/hep.28572 (2017).

6 Ozato, N. *et al. Blautia* genus associated with visceral fat accumulation in adults 20-76 years of age. *NPJ Biofilms Microbiomes* **5**, 28, doi:10.1038/s41522-019-0101-x (2019).

7 Vazquez-Moreno, M. *et al.* Association of gut microbiome with fasting triglycerides, fasting insulin and obesity status in Mexican children. *Pediatr Obes* **16**, e12748, doi:10.1111/ijpo.12748 (2021).

8 Gomez-Arango, L. F. *et al.* Low dietary fiber intake increases *Collinsella* abundance in the gut microbiota of overweight and obese pregnant women. *Gut Microbes* **9**, 189-201, doi:10.1080/19490976.2017.1406584 (2018).

9 van der Beek, C. M., Dejong, C. H. C., Troost, F. J., Masclee, A. A. M. & Lenaerts, K. Role of short-chain fatty acids in colonic inflammation, carcinogenesis, and mucosal protection and healing. *Nutr Rev* **75**, 286-305, doi:10.1093/nutrit/nuw067 (2017).

10 Sheh, A. *et al.* Alterations in common marmoset gut microbiome associated with duodenal strictures. *Sci Rep* **12**, 5277, doi:10.1038/s41598-022-09268-9 (2022).

11 Lin, Y. C., Lin, H. F., Wu, C. C., Chen, C. L. & Ni, Y. H. Pathogenic effects of *Desulfovibrio* in the gut on fatty liver in diet-induced obese mice and children with obesity. *J Gastroenterol* **57**, 913-925, doi:10.1007/s00535-022-01909-0 (2022).

12 Wan, F. *et al.* Dihydroquercetin supplement alleviates colonic inflammation potentially through improved gut microbiota community in mice. *Food Funct* **12**, 11420-11434, doi:10.1039/d1fo01422f (2021).

13 Cao, Y. G. *et al.* *Faecalibaculum rodentium* remodels retinoic acid signaling to govern eosinophil-dependent intestinal epithelial homeostasis. *Cell Host Microbe* **30**, 1295-1310 e1298, doi:10.1016/j.chom.2022.07.015 (2022).

14 Baldelli, V., Scaldaferri, F., Putignani, L. & Del Chierico, F. The role of Enterobacteriaceae in gut microbiota dysbiosis in inflammatory bowel diseases. *Microorganisms* **9**, doi:10.3390/microorganisms9040697 (2021).

15 Davin-Regli, A. & Pages, J. M. Enterobacter aerogenes and *Enterobacter cloacae*; versatile bacterial pathogens confronting antibiotic treatment. *Front Microbiol* **6**, 392, doi:10.3389/fmicb.2015.00392 (2015).

16 Li, Z. P. *et al.* Xylooligosaccharide induced changes in gut microbiota in healthy and prediabetic adults. *Faseb J* **29** (2015).

17 Rizowy, G. M. *et al.* Is the gut microbiota dysbiotic in patients with classical homocystinuria? *Biochimie* **173**, 3-11, doi:10.1016/j.biochi.2020.02.013 (2020).

18 Liu, T. H. *et al.* Gut microbiota-related evidence provides new insights into the association between activating transcription factor 4 and development of salt-induced hypertension in mice. *Front Cell Dev Biol* **8**, 585995, doi:10.3389/fcell.2020.585995 (2020).

19 Almugadam, B. S., Yang, P. & Tang, L. Analysis of jejunum microbiota of HFD/STZ diabetic rats. *Biomed Pharmacother* **138**, 111094, doi:10.1016/j.biopha.2020.111094 (2021).

20 Burakova, I. *et al.* The effect of short-term consumption of lactic acid bacteria on the gut microbiota in obese people. *Nutrients* **14**, doi:10.3390/nu14163384 (2022).

21 Nogal, A. *et al.* Circulating levels of the short-chain fatty acid acetate mediate the effect of the gut microbiome on visceral fat. *Front Microbiol* **12**, 711359, doi:10.3389/fmicb.2021.711359 (2021).

22 Wu, M. R., Chou, T. S., Huang, C. Y. & Hsiao, J. K. A potential probiotic-*Lachnospiraceae NK4A136* group: Evidence from the restoration of the dietary pattern from a high-fat diet. *Research Square*, doi:10.21203/rs.3.rs-48913/v1 (2020).

23 Li, H. *et al.* Probiotic mixture of *Lactobacillus plantarum* strains improves lipid metabolism and gut microbiota structure in high fat diet-fed mice. *Front Microbiol* **11**, 512, doi:10.3389/fmicb.2020.00512 (2020).

24 Cao, W. *et al.* The role of gut microbiota in the resistance to obesity in mice fed a high fat diet. *Int J Food Sci Nutr* **71**, 453-463, doi:10.1080/09637486.2019.1686608 (2020).

25 Lagkouvardos, I. *et al.* Sequence and cultivation study of Muribaculaceae reveals novel species, host preference, and functional potential of this yet undescribed family. *Microbiome* **7**, 28, doi:10.1186/s40168-019-0637-2 (2019).

26 Yamane, T. *et al.* Exopolysaccharides from a Scandinavian fermented milk viili increase butyric acid and *Muribaculum* members in the mouse gut. *Food Chem (Oxf)* **3**, 100042, doi:10.1016/j.fochms.2021.100042 (2021).

27 Andoh, A. *et al.* Comparison of the gut microbial community between obese and lean peoples using 16S gene sequencing in a Japanese population. *J Clin Biochem Nutr* **59**, 65-70, doi:10.3164/jcbn.15-152 (2016).

28 Serino, M. *et al.* Metabolic adaptation to a high-fat diet is associated with a change in the gut microbiota. *Gut* **61**, 543-553, doi:10.1136/gutjnl-2011-301012 (2012).

29 Wang, K. *et al.* *Parabacteroides distasonis* alleviates obesity and metabolic dysfunctions via production of succinate and secondary bile acids. *Cell Rep* **26**, 222-235 e225, doi:10.1016/j.celrep.2018.12.028 (2019).

30 Ju, T., Kong, J. Y., Stothard, P. & Willing, B. P. Defining the role of *Parasutterella*, a previously uncharacterized member of the core gut microbiota. *ISME J* **13**, 1520-1534, doi:10.1038/s41396-019-0364-5 (2019).

31 Bush, J. R. & Alfa, M. J. Increasing levels of *Parasutterella* in the gut microbiome correlate with improving low-density lipoprotein levels in healthy adults consuming resistant potato starch during a randomised trial. *BMC Nutr* **6**, 72, doi:10.1186/s40795-020-00398-9 (2020).

32 Lindberg, E. *et al.* High rate of transfer of *Staphylococcus aureus* from parental skin to infant gut flora. *J Clin Microbiol* **42**, 530-534, doi:10.1128/JCM.42.2.530-534.2004 (2004).

33 Avire, N. J., Whiley, H. & Ross, K. A review of *Streptococcus pyogenes*: public health risk factors, prevention and control. *Pathogens* **10**, doi:10.3390/pathogens10020248 (2021).

34 Aymeric, L. *et al.* Colorectal cancer specific conditions promote *Streptococcus gallolyticus* gut colonization. *Proc Natl Acad Sci U S A* **115**, E283-E291, doi:10.1073/pnas.1715112115 (2018).

35 Jiao, N. *et al.* Gut microbiome may contribute to insulin resistance and systemic inflammation in obese rodents: a meta-analysis. *Physiol Genomics* **50**, 244-254, doi:10.1152/physiolgenomics.00114.2017 (2018).
