# Supplementary material for: Common dietary emulsifiers promote metabolic disorders and intestinal microbiota dysbiosis in mice
Source: Commun Biol. 2024 Jun 20;7:749. doi: 10.1038/s42003-024-06224-3 (PMC11190199; doi:10.1038/s42003-024-06224-3)
Supplement: Supplementary file 3 — Description of Additional Supplementary Files [file 42003_2024_6224_MOESM3_ESM.pdf]

Description of Additional Supplementary Files

File name: Supplementary Data 1
Description: The source data behind the graphs in the paper.

File name: Supplementary Data 2
Description: Processed microbiome data.
